# Clinical outcomes of capitellar fractures with posterior comminution treated with Herbert screws combined with metacarpal locking plates

**DOI:** 10.1186/s12891-023-07065-7

**Published:** 2023-12-04

**Authors:** Xiang Gao, Hang Li, Deting Xue, Zhijun Pan, Yujie Zhang

**Affiliations:** Department of Orthopedic Surgery, The Second Affiliated Hospital, Zhejiang University School of Medicine, 88 Jiefang Road, Hangzhou, 310009 China

**Keywords:** Capitellar fracture, Posterior comminution, Herbert screws, Metacarpal locking plates

## Abstract

**Background:**

The treatment of Dubberley type B capitellar fractures, which are frequently complicated, is widely debated. This study aimed to investigate the prognostic factors and clinical outcomes of Dubberley type B capitellar fractures treated with Herbert screws combined with posterior buttress plates.

**Methods:**

Seven men and nine women (aged 30–68 years) with Dubberley type B capitellar fractures were operated on with Herbert screws combined with posterior buttress plates. The patients were classified into Dubberley types IB (seven), IIB (four), and IIIB (five). Complications and bone union were observed, and functional outcomes were evaluated by the Mayo Elbow Performance Index (MEPI).

**Results:**

All patients were followed up for a mean period of 23.5 months (12–30 months). All fractures healed in 8–14 weeks (mean, 10.5 weeks). No cases of non-union, elbow instability, or avascular necrosis occurred. Degenerative arthritis occurred in 7 (44%) and heterotopic ossification in 11 (69%) patients. The median MEPI score was 92.5 (interquartile range, 85–100) points, with 11 reporting excellent, 3 good, and 2 fair outcomes. The MEPI scores of type IIIB fractures were significantly lower than those of types IB and IIB fractures, while the MEPI scores of type IB and IIB fractures did not differ significantly.

**Conclusions:**

Dubberley type IIIB capitellar fractures with multiple articular fragments have a poorer prognosis than type IB and IIB fractures. However, Herbert screw fixation combined with posterior metacarpal locking plates is feasible, providing satisfactory recovery of elbow joint function.

**Supplementary Information:**

The online version contains supplementary material available at 10.1186/s12891-023-07065-7.

## Background

Capitellar fractures, which involve the capitellum with or without fractures of the trochlea, are relatively uncommon and comprise only 0.5–1% of all elbow fractures [1, 2]. The capitellum forms the anterior and inferior surfaces of the distal humerus at the elbow joint. Loss of the sphericity of the capitellum may markedly impair the elbow’s range of motion [3]. Furthermore, because of a paucity of soft tissue attachments at this site, such fractures often result in free articular fragments that may become displaced.

The classification of capitellar injuries has evolved from initially descriptive systems with no prognostic significance to more advanced systems with the capacity to direct treatment strategy and predict prognosis based on multi-planar imaging [4–6]. Dubberley et al. put forward a treatment- and outcome-oriented system which considers trochlear involvement and posterior comminution [7]. Type I fractures of the capitellum may or may not involve the lateral trochlear ridge. Type II fractures are single fragment fractures of the capitellum and trochlea, while type III are the capitellum and trochlea broken into separate fragments. The fractures are further classified into subtypes A or B depending on the absence or presence of posterior comminution. The Dubberley system has been widely used in clinical practice because of its relation to prognosis, wherein higher-grade fractures show worse outcomes.

Dubberley type B capitellar fractures are commonly complicated by the presence of posterior comminution, impacted articular fragments, and limited bone stock for internal fixation [8]. To date, the management of Dubberley type B capitellar fractures remains unelucidated. Moreover, because of the rarity of Dubberley type B fractures, most orthopedic surgeons have limited experience in its treatment. Therefore, this retrospective study aimed to outline the surgical technique of open reduction and internal fixation (ORIF) for treating Dubberley type B capitellar fractures and explore its clinical and functional results to improve the management of capitellar fractures.

## Methods

### Inclusion or exclusion criteria

Inclusion criteria were as follows: 1) adults > 18 years old; 2) patients with distal humeral fractures of type 13-B3.1 and type B3.3 diagnosed using computed tomography (CT) as comminution of the posterior humerus; 3) those with complete follow-up data and a follow-up duration of more than 12 months.

Exclusion criteria were as follows: 1) patients with 13-C distal humerus fracture; 2) patients with rheumatoid arthritis and other bone and joint systemic diseases; 3) patients with mental and psychological diseases who cannot cooperate with treatment and rehabilitation; 4) those with incomplete follow-up data and follow-up duration less than 12 months.

### Patients

**Fig. 1:**
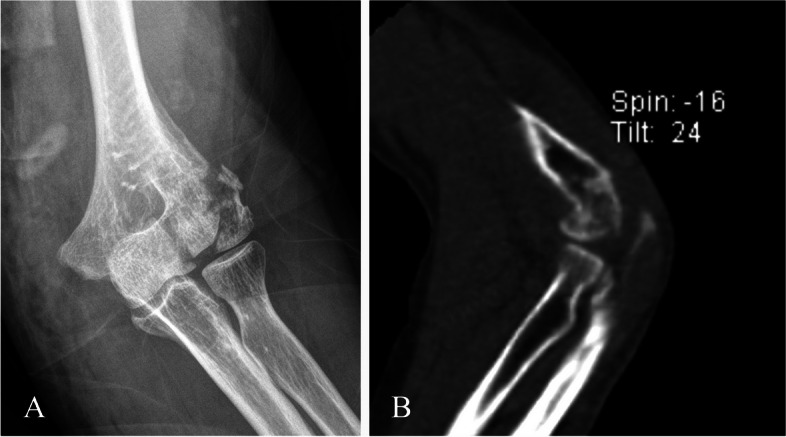
A type IIIB capitellum fracture in a 61-year-old woman who fell on an outstretched hand. **A** Preoperative anteroposterior radiograph and **B** sagittal computed tomography scan

All patients with capitellar fractures with posterior condylar comminution (Dubberley classification B type) treated by ORIF in our hospital between May 2013 and September 2016 were enrolled in the study. The diagnosis was made and confirmed by the elbow radiographs in the anteroposterior and lateral views and preoperative computed tomography (CT) (Fig. 1).

This study was approved by The Human Research Ethics Committee of The Second Affiliated Hospital, Zhejiang University School of Medicine, and performed in accordance with the Declaration of Helsinki. Informed consent was obtained from all patients.

### Surgical technique

After induction of general anesthesia, the ligamentous stability of the injured elbow was assessed clinically. An extensive lateral approach was performed, proceeding cautiously to leave the lateral ulnar collateral ligament (LUCL) intact. The release of the ligament is performed only when a satisfactory exposure is needed to get an anatomical reduction of the articular surface.

**Fig. 2:**
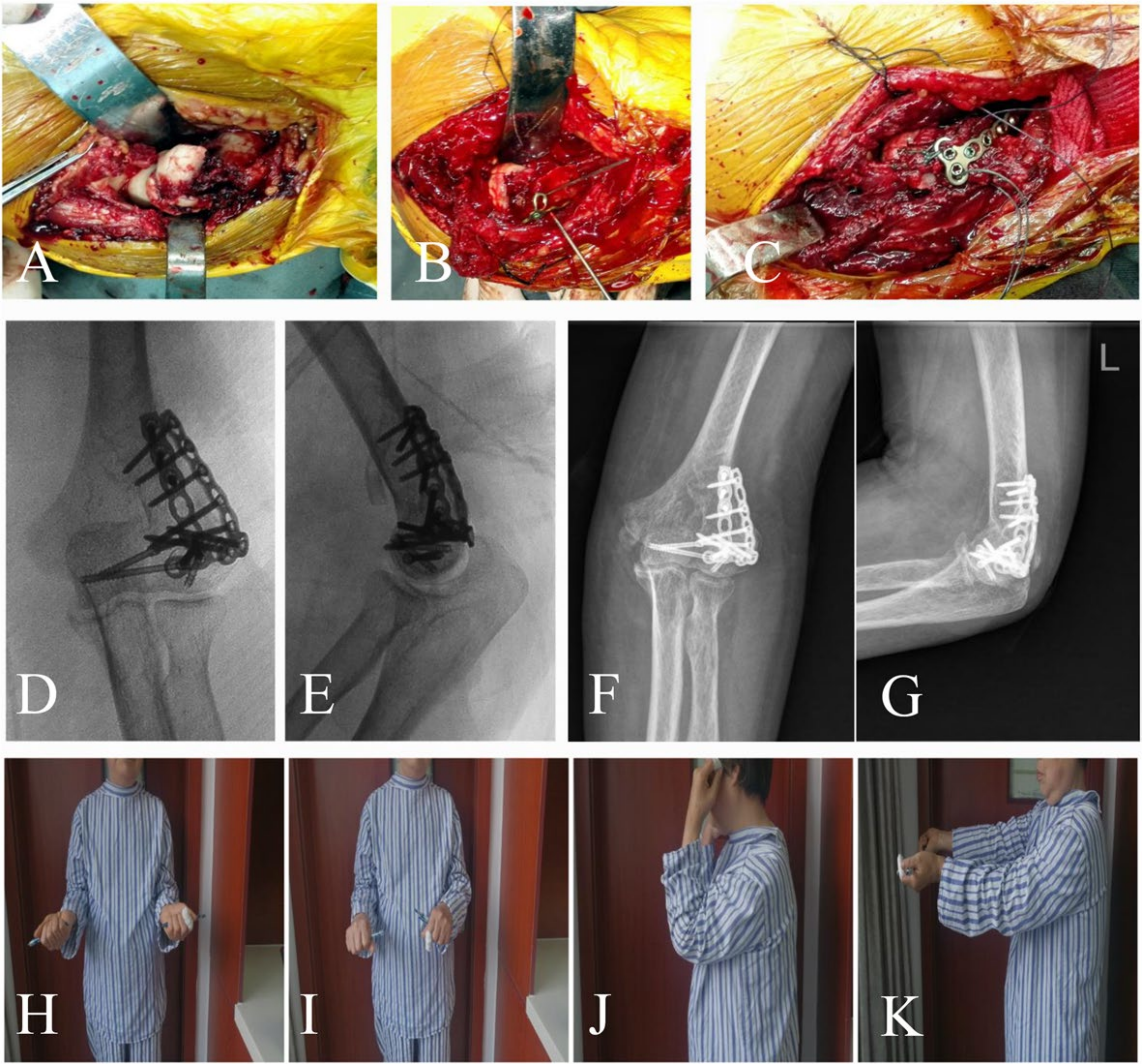
Surgical exposure and fixation of the same patient as in Fig. 1. **A** A type IIIB fracture is exposed by the extensive lateral approach. The epicondylar fragment with the lateral ulnar collateral ligament is reflected distally to enhance exposure. **B** Reconstruction of the lateral column is performed with a 2.0 mm metacarpal locking plate. A tricortical iliac crest bone autograft is used to bridge the bone defect. **C** The lateral ulnar collateral ligament is repaired to its origin using a figure-of-eight tension band wire technique. **D**, **E** Intraoperative radiological outcome. **F**, **G** X-ray plain film at 18 months after surgery. **H**–**K** Mild limitation of elbow flexion and pronation at 18 months of follow-up; the Mayo Elbow Performance Index is 90 points

This study performed the reduction and fixation of the segments in sequential order. In the case of lateral trochlear involvement, this fragment was reconstructed first, allowing for appropriate anatomical positioning of the laterally subluxated ulna. The posterolateral portion of the distal humerus was then reconstructed, and a pre-contoured 2.0 mm metacarpal locking plate (Synthes, Soletta, Switzerland) was used to buttress the comminuted posterolateral portion. A tricortical iliac crest bone autograft harvest was used to bridge the posterolateral bone defect where necessary (Fig. 2). Subsequently, reduction and stabilization of the capitellar segments were performed. Impacted segments were gently elevated, and supplemental autologous bone grafting was harvested to fill the metaphyseal defect. Finally, the LUCL was repaired to its origin by suture anchors or a figure-of-eight tension band in the case of avulsion or bone fragments.

**Fig. 3:**
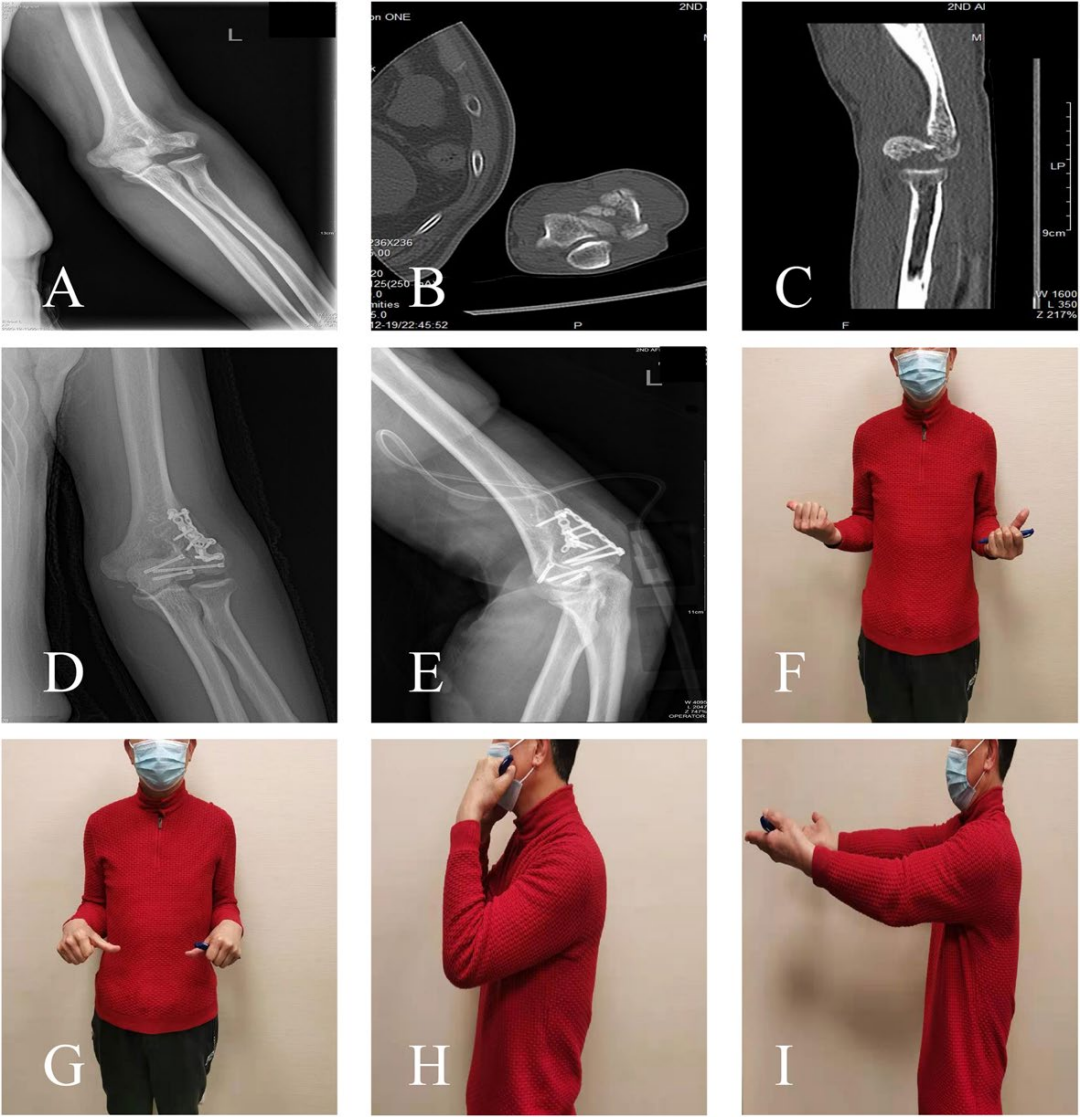
A type IIIB capitellum fracture in a 68-year-old male patient. **A** Preoperative anteroposterior radiograph and **B**, **C** CT scan. **D**, **E** X-ray plain film at 12 months after surgery. **H**–**K** Mild limitation of elbow flexion and pronation at 12 months of follow-up; the Mayo Elbow Performance Index is 90 points

Bone fragments were reduced and temporarily fixed with K-wires. Headless compression screws (Synthes) were placed from anterior to posterior to avoid further disrupting the remaining vascular supply. A minimum of two screws were placed to ensure rotational stability (Fig. 3). After fixation, the elbow was fully pronated and supinated under fluoroscopy to assess joint stability and consistency. Next, the common extensors were repaired to their origin at the supracondylar ridge. Subcutaneous closure was carried out layer by layer. Then, the elbow was splinted at 90°.

### Postoperative management

The splint was removed 3 days after surgery. According to patient tolerance, full range-of-motion exercises were initiated under adequate analgesia (tramadol 50 mg twice daily for 7–14 days). A home exercise program was prescribed as follows: first 2 weeks, full range-of-motion exercises under the protection of a hinged elbow braces; subsequently, the forearm rotation exercise can be included in the agenda; elbow radiographs and range of motion were assessed at 4 weeks; after 12 weeks, check for fracture healing and gradually start upper limb weight-recovery function training if healing was good. Our treatment regimen did not include heterotrophic ossification prophylaxis. All patients were followed up with a regular clinical and radiographic evaluation. The functional assessment was performed using the Mayo Elbow Performance Index (MEPI) at the last follow-up [9]. Degenerative arthritis (DA) was scaled based on the Broberg and Morrey system, and heterotopic ossification (HO) was stratified by the Brooker system used in the elbow [10, 11]. We also collected the incidence of elbow-related complications, such as nonunion of the bone, wound healing problems, and avascular necrosis on radiographs, which were recorded.

### Statistical analyses

Statistical analyses were performed on SPSS 19.0 software. Significant differences were analyzed using the Kruskal–Wallis test and the Mann–Whitney U test. Correlations between two datasets were estimated using Spearman’s rank correlation test. A value of *P*-value of < 0.05 was considered significant.

## Results

Between May 2013 and September 2016, 16 patients (7 men and 9 women) aged 30–68 years (mean, 47 years) with closed capitellar fractures combined with posterolateral condylar comminution were treated with Herbert screws and metacarpal locking plates. The mechanism of injury included 3 patients who had a motor vehicle incident, a fall in 10, and other causes (unknown) in 3. Concomitant injuries included distal radius fractures in three patients, a radius head fracture in one, and a scaphoid fracture in one. The right and left elbows were involved in every eight cases. Seven patients were categorized as Dubberley type IB, type IIB (four patients), and IIIB (five). The main features of included patients are summarized in Table 1.

**Table 1:** Epidemiologic data, fracture type, and functional outcomes

	Type	Age	Sex	Time to surgery (days)	MEPI	DA	HO	Side	Follow-up (months)	Extension (°)	Flexion (°)
1	IB	30	M	14	100	0	1	L	14	0	135
2	IB	42	F	23	95	1	1	L	30	10	130
3	IB	53	F	6	100	0	0	L	35	0	130
4	IB	32	M	7	100	0	2	R	30	10	130
5	IB	40	F	9	85	0	1	L	40	20	110
6	IB	42	M	9	100	1	1	L	22	15	135
7	IB	41	M	10	85	0	0	R	14	25	120
8	IIB	53	F	7	90	1	1	R	29	20	125
9	IIB	32	M	5	100	0	0	L	14	15	130
10	IIB	54	F	7	95	1	1	R	27	20	125
11	IIB	64	F	12	100	0	0	R	22	10	130
12	IIIB	68	M	6	90	0	1	L	12	20	130
13	IIIB	61	F	17	80	1	2	L	29	30	110
14	IIIB	40	M	8	65	0	1	R	19	35	105
15	IIIB	50	F	16	90	1	1	R	24	30	130
16	IIIB	56	F	7	70	1	0	R	15	35	100

*MEPI* Mayo Elbow Performance Index, *DA* degenerative arthritis, *HO* Heterotopic ossification, *M* Male, *F* Female, *L* Left, *R* Right

The mean operation time was 96 min (55–143 min). No intraoperative complications were encountered. The LUCL was found to be damaged with small lateral epicondylar avulsion fragments in 5 of 16 elbows. No cases were found of LUCL avulsion at its point of attachment to the lateral epicondyle or rupture at its mid-substance region. A tricortical iliac crest bone autograft harvest was used in 2 of 16 patients to bridge the extensive comminution of the lateral column.

**Fig. 4:**
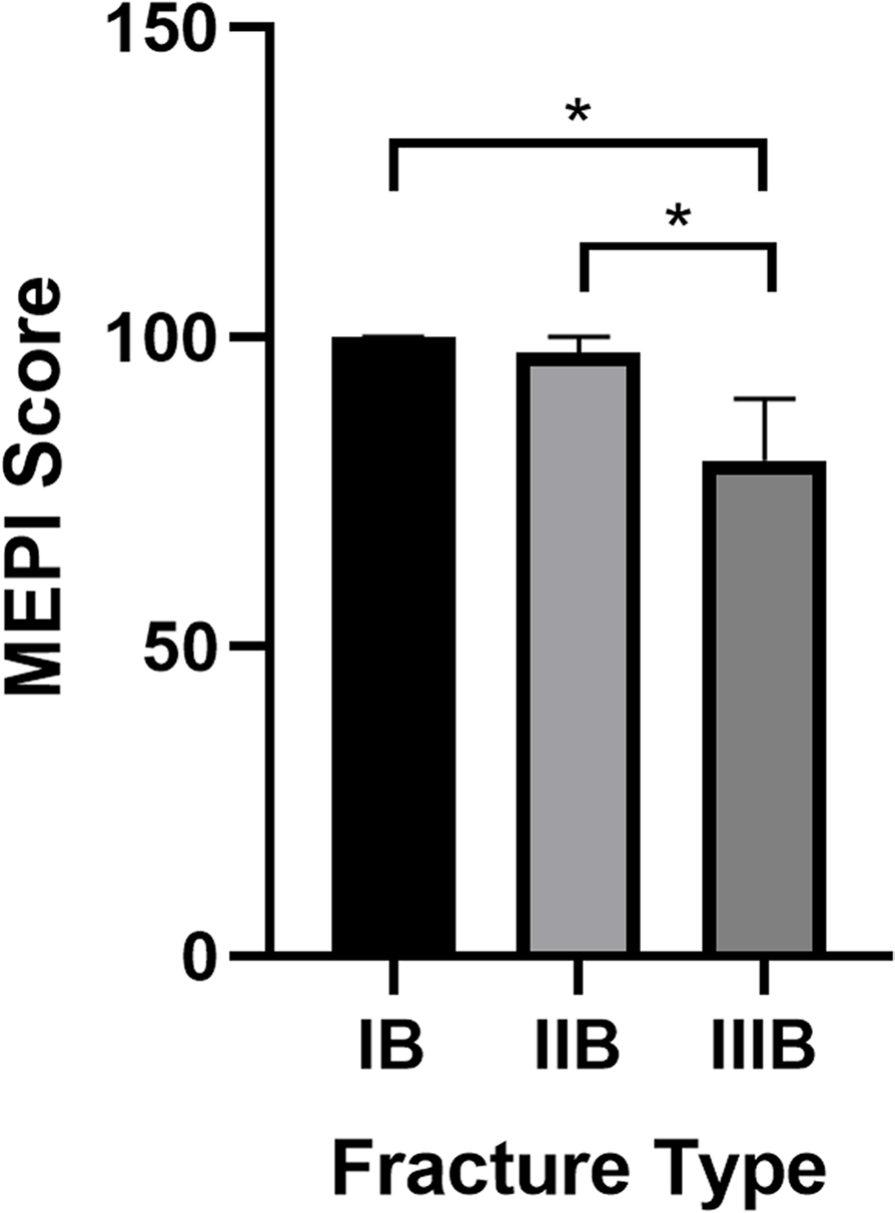
The MEPI score of three types of fractures

The average follow-up time was 23.5 months (12–40 months). Outcomes were graded as excellent (MEPI ≥ 90) in 11 patients, good (75 ≤ MEPI <90) in 3, and fair (MEPI <75) in 2. The mean range of flexion and extension were 123.4°±10.7° (range, 100°–135°) and 18.5°±10.1° (range, 0°–35°), respectively. All patients returned to their normal occupations and hobbies, such as gardening, bicycle riding, and photography. As for their functional recovery, the median MEPI score of type IB fractures was 100 points (IQR 85–100), 92.5 (IQR 85–100) for type IIB, and 80 (IQR 67.5–90) for type IIIB at the last follow-up (Fig. 4). The MEPI scores differed significantly between the three types of fractures (Kruskal–Wallis test, *P* = 0.031), with that of type IIIB fractures significantly lower than those of type IB and IIB fractures (Mann–Whitney U test, *P* = 0.025 and *P* = 0.024, respectively) (Fig. 4). In addition, no obvious difference between the MEPI scores of type IB and IIB fractures was noted (Mann–Whitney U test, *P* = 0.917). Age, sex, and the time from injury to the operative intervention were not associated with MEPI scores (Table 2) (*P* > 0.05).

All the incision wounds healed uneventfully. No cases of wound infection, non-union, avascular necrosis, or elbow instability occurred. The complications were degenerative arthritis in 7 (44%) and heterotopic ossification in 11 (69%). DA and HO were not associated with MEPI scores (Mann-Whitney U test, *P* = 0.382 and *P* = 0.599, respectively).

**Table 2:** Associations between demographic and clinical variables on functional outcomes

Variable	Statistical method	*P* value
Sex	Mann–Whitney U	0.445
Age	Kruskal–Wallis test	0.246
Time from injury to surgical intervention	Spearman’s rank correlation test	0.915

## Discussion

Treatment options for capitellar fractures vary from non-surgical treatment, fragment excision, and ORIF to total elbow arthroplasty [12, 13]. ORIF is considered the preferred modality for treating capitellar fractures [14]. The main goal of surgical therapy is to restore articular congruity and maintain stable fixation, allowing for early and full rehabilitation [3, 15]. However, the rehabilitation goals may be technically difficult to reach, especially in the presence of substantial comminution, bone loss, or osteoporosis [16]. Studies have demonstrated that Dubberley type B capitellar fractures are often associated with compromised vascular supply to the fragments, which can negatively impact the functional outcome [8]. In our case series, we used Herbert screw fixation combined with a metacarpal locking plate to treat Dubberley type B capitellar fractures with satisfactory results.

In our cases, we used extended EDC splitting approach, which employs a more anterior position than the Kocher approach. It can provide greater and more reliable exposure of coronal fracture fragment, and is beneficial for reduction of capitellar fractures [17]. Besides, this approach preserves the origin of the ulnar extensor carpal muscle and the posterior half of the origin of the common extensor finger, reducing the risk of iatrogenic injury to the lateral ulnar collateral ligament and causing posterolateral rotational instability, while preserving as much blood supply as possible to the capitellum [18].

It is very difficult to reduce the Dubberley type B fracture, due to factors such as compression of the articular surface and osteoporosis. Herein, we proposed a treatment algorithm for Dubberley type B capitellar fractures. In this group of cases, the trochlear fragment was first reduced, after which the posterior lateral condyle of the humerus was fixed with the posterior buttress plate. Finally, the capitellum and the external ulnar collateral ligament were reduced and fixed. The trochlear fragment was relatively complete, often presenting with simple split fracture, facilitating easy anatomic reduction. Prior fixation of the trochlear fragment helps to more effectively determine the bone involution relationship of the elbow, and provides reference for reduction and fixation of the articular surface of the capitellum [18].

The presence of posterior comminution caused instability of the fracture fixation, wherein a posterior plate may be required to buttress the lateral column [19]. Plates used for buttressing the lateral column reported in the literature include pelvic reconstruction plates, distal humerus anatomic plates, and pre-contoured distal humerus locking plates [15, 20]. All the plates mentioned above have difficulty fixing small fragments together due to relatively large screw diameter and screw hole span width. Furthermore, these plates may irritate the ligaments and soft tissues, impeding early and full rehabilitation [3]. This present study elected to use 2.0 mm metacarpal locking plates to reconstruct the posteroinferior part of the distal lateral column because of their smaller screw diameter, screw hole span width, and ease of contouring in three dimensions to match the anatomy. The locking design can increase construct stability and decrease the risk of screw back-out and subsequent loss of reduction. Furthermore, they have the advantage of minimal irritation of ligaments and soft tissue due to their low-profile design. We propose that the 2.0 mm metacarpal locking plate is the ideal implant currently available for the reconstruction of the lateral column that allows enough stability for early movement.

In this study, 9 of the 16 fractures occurred in women, which displayed a similar trend to other studies, ranging from 60 to 90% female dominance. This trend could result from poorer bone mineral density and an increased carrying angle than that of men [2, 7, 21]. Capitellar fractures can occur in all age groups. Our study included patients aged 30–68 years and showed no significant correlation between age and functional outcomes. Some studies reporting outcomes following ORIF of capitellar fractures have demonstrated that posterior comminution of the lateral condyle is more likely to result in a poor outcome [8]. However, the available literature on the association between functional outcome and fracture subtypes of Dubberley type B fractures is limited. In the current study, no statistical difference was observed in MEPI scores between type IB and IIB fractures. Our study indicates that multiple articular fragments are more likely to compromise the final outcome. Accordingly, type IIIB fractures exhibited lower MEPI scores than type IB and IIB fractures.

Stiffness is a complication that is commonly encountered following all elbow injuries. A prolonged period of immobilization may have contributed to elbow stiffness and poor functional results [22]. Early and full range-of-motion exercises are essential to a good functional outcome, and this can only be performed with effective analgesia. In this series, patients received tramadol 50 mg twice daily to facilitate a full range of motion exercises. No patient in the series showed any evidence of instability, notwithstanding early and full mobilization. Osteonecrosis of the capitellum and/or trochlea is another complication following elbow injuries, with a 0–30% reported incidence [23, 24]. It may be associated with capitellar displacement and rotation, separation from soft-tissue attachments, and posterior metaphyseal comminution. None of the patients in our study developed osteonecrosis or avascular necrosis, and the union was achieved in all patients. Mild to moderate heterotopic ossification and posttraumatic osteoarthrosis may be observed at intermediate follow-up. In the present study, the incidence of posttraumatic osteoarthrosis was 44%, and that of heterotopic ossification was 69%. No cases of clinically significant heterotopic ossification were observed in our study. Therefore, no concrete evidence exists on the need for prophylactic treatment of heterotopic ossification.

This study has limitations. First, it was designed retrospective in nature. Second, it had a small sample size. However, owing to the low incidence of this injury, our results may still contribute to the limited available literature on Dubberley type B fractures. Furthermore, prospective studies using larger cohorts are needed to confirm our findings.

## Conclusions

Dubberley type IIIB capitellar fractures are more likely to have poor outcomes. Treatment with ORIF with Herbert screws and 2.0 mm posterior metacarpal locking plates maintains a stable fixation and obtains satisfactory clinical results in patients with capitellar fractures with posterolateral comminution.

### Supplementary Information

**Additional file 1: Figure 1.** A type IIIB capitellum fracture in a 56-year-old female patient. (A) Preoperative anteroposterior radiograph and (B, C) CT scan. (D) X-ray plain film at 15 months after surgery.

## Data Availability

All data relevant to the study are included in the article or are available from the corresponding author on reasonable request.

## Abbreviations

CT: Computed tomography
DA: Degenerative arthritis
HO: Heterotopic ossification
LUCL: Lateral ulnar collateral ligament
MEPI: Mayo Elbow Performance Index
ORIF: Open reduction and internal fixation
