# Supplementary material for: Clinical outcomes of capitellar fractures with posterior comminution treated with Herbert screws combined with metacarpal locking plates
Source: BMC Musculoskelet Disord. 2023 Dec 4;24:937. doi: 10.1186/s12891-023-07065-7 (PMC10694941; doi:10.1186/s12891-023-07065-7)
Supplement: Supplementary file 1 — Additional file 1: Figure 1. A type IIIB capitellum fracture in a 56-year-old female patient. (A) Preoperative anteroposterior radiograph and (B, C) CT scan. (D) X-ray plain film at 15 months after surgery. [file 12891_2023_7065_MOESM1_ESM.docx]

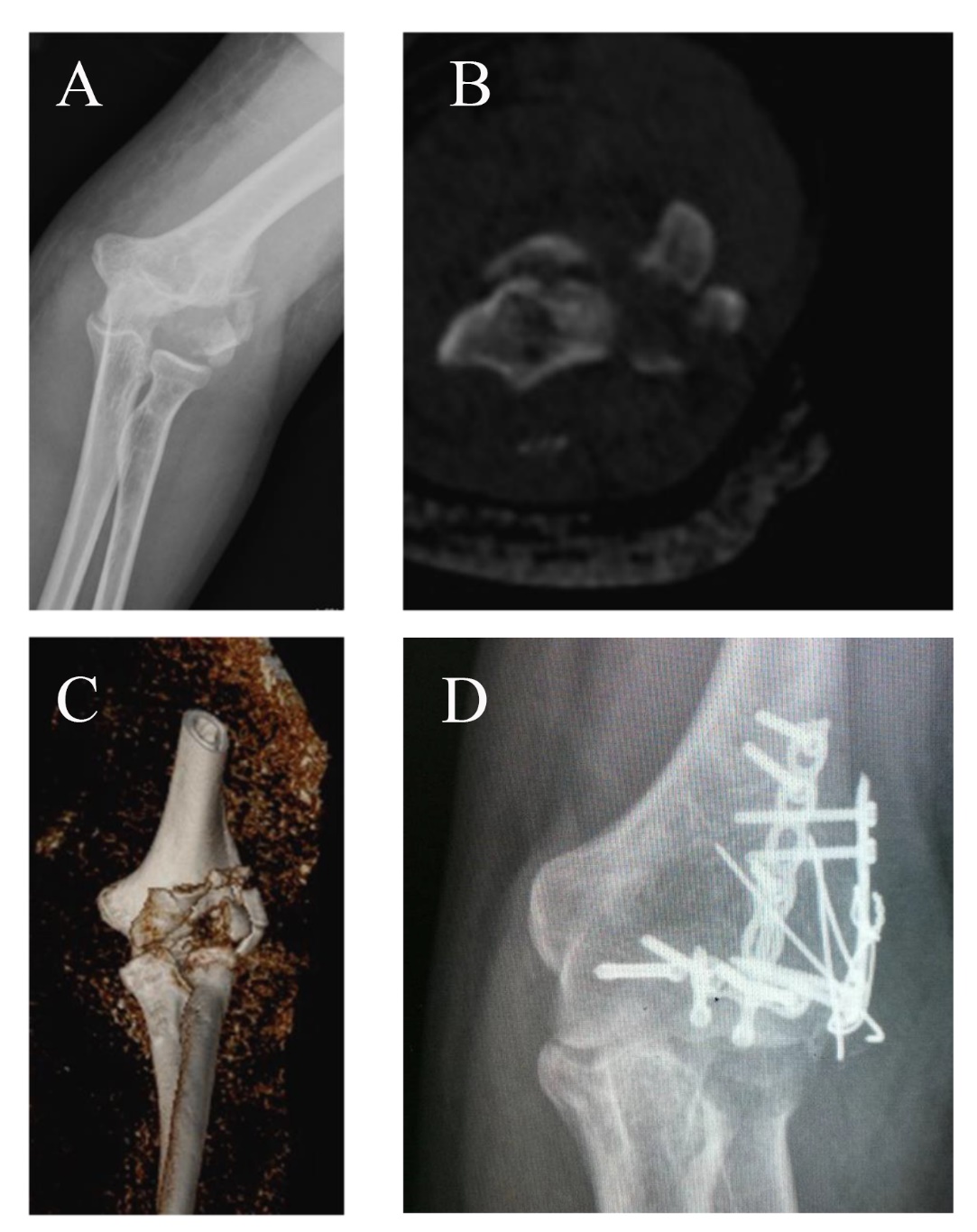


**Figure 1** A type IIIB capitellum fracture in a 56-year-old female patient. (A) Preoperative anteroposterior radiograph and (B, C) CT scan. (D) X-ray plain film at 15 months after surgery.
